# Latent profile analysis of postpartum depression symptoms and its associations with weight stigma and eating behaviors among postpartum women in China

**DOI:** 10.1186/s40359-026-05303-w

**Published:** 2026-08-11

**Authors:** Tian Xu, Jiayuan Peng, Xiangmin Tan, Yuxian Huang, Yuqing He, Yi Zeng, Jingfei Tang, Rongzhi Li, Lai Peng, Yuan Zeng, Mei Sun

**Affiliations:** 1 Xiangya School of Nursing, Central South University, 172 Tongzipo Road Yuelu district, Changsha, 410013 Hunan China; 2 The Affiliated Hospital of Shandong University of Traditional Chinese Medicine, Jinan, China; 3 Xiangyin Campus of Xiangxing College, Hunan University of Chinese Medicine, Yueyang, China; 4 School of Rural Health, Monash University, 15 Sargeant Street, Warragul, Victoria 3820 Australia; 5 School of Clinical Sciences at Monash Health, Monash University, Clayton, Victoria Australia; 6 School of Nursing, Changsha Medical University, Changsha, China; 7 Department of Orthopedics, Movement System Injury and Repair Research Center, Xiangya Hospital, Central South University, Changsha, China; 8 The Fourth Affiliated Hospital of Xinjiang Medical University, 116 Huanghe Road, Shaybak District, Urumqi, 830000 Xinjiang Uygur Autonomous Region China

**Keywords:** Postpartum depression, Latent profile analysis, Weight stigma, Weight bias internalization, Eating behaviors

## Abstract

**Background:**

Postpartum depression (PPD) represents a significant maternal health challenge with diverse symptom manifestations. Traditional analytical approaches that treat postpartum women as a homogeneous group may obscure distinct symptom patterns, limiting the development of targeted nursing interventions.

**Objectives:**

This study aimed to identify distinct subgroups of Chinese postpartum women based on their depression symptom patterns using latent profile analysis, and to examine factors associated with subgroup membership, with particular focus on weight-related stigma and eating behaviors.

**Methods:**

In this cross-sectional study, we recruited 507 postpartum women within one year of delivery between December 2021 and March 2022. Participants completed standardized measures including the Edinburgh Postnatal Depression Scale (EPDS), Perceived Weight Stigma Questionnaire (PWSQ), Weight Bias Internalization Scale (WBIS), and Dutch Eating Behavior Questionnaire (DEBQ). Latent profile analysis (LPA) was conducted using the 10 EPDS items, with multinomial logistic regression employed to identify factors associated with profile membership.

**Results:**

LPA revealed three distinct subgroups, characterized primarily by symptom severity: ‘low-level PPD’ (45.2%), ‘middle-level PPD’ (41.8%), and ‘high-level PPD’ (13.0%). Factors significantly associated with profile membership included perceived weight stigma, weight bias internalization, emotional eating, external eating, age, body mass index, and education level.

**Conclusions:**

The identification of three distinct PPD profiles highlights the heterogeneity in depression symptom presentation among Chinese postpartum women. These findings support the adoption of person-centered assessment approaches in nursing practice. Screening for weight-related stigma and maladaptive eating behaviors may enhance early identification of high-risk women. The results provide empirical evidence for developing tailored nursing interventions that address profile-specific characteristics, offering a step toward precision in postpartum mental healthcare.

## Introduction

Postpartum depression (PPD) is a substantial public health challenge, characterized by depressive symptoms occurring within one year after childbirth [1]. Affecting 17–22% of women globally [2], PPD represents one of the most common perinatal mental health conditions. In China, its prevalence reaches at least 20% and continues to rise [3, 4].

PPD imposes severe impacts on postpartum women, their families and society. It causes low mood, altered eating patterns and reduced self-efficacy, impairing maternal physical and mental health as well as quality of life [5, 6]. Notably, 37.9% of symptomatic women report suicidal thoughts in the preceding week, and some even contemplate infant harm [7, 8]. Children of affected mothers also face higher risks of cognitive developmental deficits, behavioral problems and social adjustment difficulties [9].

As the world’s largest developing country, China faces unique challenges in addressing PPD. Socioeconomic factors such as higher unemployment and lower income levels contribute to its elevated prevalence [2]. A meta-analysis of 565 studies across 80 countries revealed PPD prevalence in developing nations (19.99%) is significantly higher than in developed countries (14.85%) [2]. Culturally, deep-rooted son preference [10], and the idealization of the ‘resilient, self-sacrificing perfect mother’ further burden postpartum women [11].

The WHO has prioritized mental health in maternal care through universal health coverage goals [12], emphasizing recognition of women’s needs and cultural context. Similarly, China’s National Health Commission (NHSC) launched the Exploration of Special Service Work Plan for Depression Prevention and Treatment, highlighting PPD’s urgency [13].

Previous studies have identified associations between PPD and sociodemographic characteristics (e.g., body mass index [BMI] [14]), and psychosocial factors (e.g., eating behaviors [15], weight stigma [16]). It is important to note that the Edinburgh Postnatal Depression Scale (EPDS), the most commonly used tool in PPD research, is a screening instrument designed to identify probable cases of PPD rather than a diagnostic tool for clinical depression. Weight stigma refers to weight-based social prejudice, discrimination, negative evaluation or unfair treatment [17], and can affect even women with ‘normal’ BMI [18]. Weight bias internalization—its internalized form—involves adopting societal weight-based negative stereotypes into self-criticism and negative self-evaluation [19]. To our knowledge, only two Chinese studies have explored the weight stigma among postpartum women [18, 20], but none has examined how these factors differentiate distinct PPD symptom subgroups.

The postpartum period is uniquely vulnerable to weight-related issues [16], shaped by conflicting cultural norms. Traditional ‘sitting the month (zuò yuèzi)’ is a core practice involving reduced physical activity, increased nutritious intake, rest and breastfeeding to facilitate recovery [21], where weight gain is viewed as a sign of adequate nourishment [22]. However, modern China’s growing emphasis on the ‘thin ideal’ has intensified weight-based prejudice [23], creating a unique psychological conflict for postpartum women. Regarding eating behaviors, three distinct types are examined: emotional eating (triggered by negative emotions), external eating (driven by external cues like food aroma), and restrained eating (deliberate restriction for weight control) [24]. While these behaviors are linked to PPD [15], few studies have explored their variability across distinct PPD symptom subgroups [20].

Notably, most PPD research relies on a “variable-centered” approach, using aggregate EPDS scores or threshold cut-offs to classify participants into binary ‘depressed’ versus ‘non-depressed’ groups [6, 25, 26]. This approach has critical limitations: it assumes homogeneity in symptom expression and variable relationships, overlooks distinct symptom profiles among women with similar total scores [27]. In contrast, person-centered approaches such as latent profile analysis (LPA) identify subgroups with shared symptom patterns, thereby capturing population heterogeneity that is obscured by variable-centered methods [28]. International LPA studies consistently identify 3–4 PPD subgroups, often differentiated by emotional dysregulation or mixed symptoms [29]. While a few Chinese LPA studies have explored postpartum adverse symptom profiles or maternal PPD literacy subtypes [30], none has specifically focused on PPD symptom profiles in relation to weight stigma and eating behaviors.

This study aims to address these gaps by (a) identifying and describing PPD symptom profiles among Chinese postpartum women using LPA based on EPDS items; and (b) analyzing factors associated with profile membership. As one of the first applications of LPA in this population, this study provides a person-centered framework for identifying subgroups with distinct associated characteristics, which may ultimately inform the development of tailored interventions to address the diverse needs of postpartum women.

## Methods

### Participants

This cross-sectional study was conducted in accordance with the STROBE checklist for observational studies [31]. Data were collected from December 2021 to March 2022 in Changsha, China. Participants were recruited through convenience sampling from postpartum follow-up waiting areas of Changsha Maternity and Child Health Hospital (a public urban tertiary hospital) and four public urban community health service centers. Trained research staff explained the study objectives, voluntary and anonymous nature of participation, and absence of potential harm to eligible participants. Those who provided both verbal and written informed consent were given paper questionnaires to complete in a designated private room, with on-site assistance available if needed. The average completion time was 10–15 min, and completed questionnaires were collected immediately.

To be eligible for the study, participants had to meet the following criteria: (a) women of childbearing age (18–49 years) who were between one week and one year postpartum; (b) ability to communicate effectively; (c) ability to read and complete questionnaires independently (at least primary school education level); and (d) voluntary agreement to participate with written informed consent. Exclusion criteria included: (a) individuals with impaired consciousness or diagnosed psychiatric disorders; and (b) those currently receiving psychological treatment.

The sample size was determined based on two key considerations: (1) LPA requires a minimum of 500 participants to ensure stable model fitting [32]; (2) multinomial logistic regression requires 10–20 participants per independent variable [49]. This study included 8 independent variables (age, BMI, education level, PWSQ, WBIS, emotional eating, external eating, restrained eating), requiring a minimum sample size of 80–160. A total of 530 postpartum women were invited to participate, with 23 excluded due to incomplete questionnaires or missing key data, resulting in 507 valid samples.

Convenience sampling was employed due to feasibility considerations. Consequently, the sample may not be fully representative of the broader postpartum population in China, particularly regarding the overrepresentation of urban residents (68.2%) and women with higher education levels (70.6% held a university degree or above).

### Measures

#### Demographic characteristics

Socio-demographic variables collected in this study included age, residence, education level, monthly income (RMB), number of births, and type of childbirth.

#### Perceived Weight Stigma Questionnaire (PWSQ)

Perceived weight stigma is defined as the subjective experience of being discriminated against, stereotyped, or treated unfairly by others due to one’s body weight [33]. The PWSQ is a 10-item self-report scale that assesses the frequency of such experiences. Each item is scored dichotomously (‘yes’=1, ‘no’=0), with total scores ranging from 0 to 10; higher scores indicate more frequent perceived weight stigma. The Chinese version of the PWSQ has demonstrated acceptable psychometric properties in Chinese populations [34, 35]. In the current sample, the PWSQ exhibited a Cronbach’s α of 0.80, indicating adequate reliability.

#### Weight Bias Internalization Scale (WBIS)

Weight bias internalization refers to the process of internalizing societal weight-based negative stereotypes and converting them into self-criticism, self-blame, and negative self-evaluation [19]. The WBIS, developed by Durso and Latner [36], measures the degree of internalized weight stigma using 11 items rated on a 5-point Likert scale (1 = strongly disagree to 5 = strongly agree). Higher scores reflect greater internalization of weight bias. The scale has demonstrated good internal consistency, with a Cronbach’s α of 0.87 among overweight or obese German adolescents [37], and has been successfully validated in Chinese children and adolescents [38]. More recently, the Chinese version of the WBIS has shown satisfactory psychometric properties in Chinese postpartum women [35]. In the current study, its Cronbach’s α was 0.90, indicating excellent reliability.

#### Edinburgh Postpartum Depression Scale (EPDS)

The EPDS, developed by Cox and Holden [39] and adapted into Chinese by Lee and Yip [40], was used to assess postpartum depressive symptoms. This 10-item scale uses a 4-point Likert format (0 = not at all to 3 = yes, nearly every day), with total scores ranging from 0 to 30. A cut-off score of ≥ 13 is widely used to identify probable PPD in Chinese populations [40]. It is important to note that the EPDS is a screening tool designed to identify probable cases of PPD, rather than a diagnostic instrument for clinical depression. Higher scores indicate more severe depressive symptoms. In the current study, the Chinese version’s Cronbach’s α was 0.90, indicating strong internal reliability.

Prior to conducting LPA, the study examined the internal consistency of the EPDS items, which demonstrated acceptable reliability (Cronbach’s α = 0.90).

#### Dutch Eating Behavior Questionnaire (DEBQ)

The DEBQ, developed by van Strien [41], assesses three distinct maladaptive eating behaviors. Emotional eating refers to eating triggered by negative emotions rather than internal hunger cues. External eating is eating driven by external environmental cues independent of hunger. Restrained eating involves deliberate restriction of food intake to control body weight or shape. The scale comprises 33 items across the three subscales (13, 10, and 10 items respectively). Items are rated on a 5-point scale (1 = never to 5 = very often), with higher subscale scores indicating greater levels of the corresponding eating behavior. The Chinese version of the DEBQ has been validated in Chinese populations and demonstrated good internal consistency [42]. In this study, Cronbach’s α for emotional, external, and restrained eating were 0.96, 0.89, and 0.93, respectively, reflecting excellent internal consistency.

### Data analysis

Descriptive statistics were computed using SPSS, version 29.0. Categorical variables were presented as frequencies and percentages, while continuous variables were reported as mean ± standard deviation (M ± SD).

LPA was conducted in Mplus, version 8.3, to identify subgroups of PPD based on the EPDS. Model fit was evaluated using several indices: Akaike Information Criterion (AIC), Bayesian Information Criterion (BIC), adjusted Bayesian Information Criterion (aBIC), Entropy, Lo-Mendell-Rubin (LMR) test, and the Bootstrapped Likelihood Ratio Test (BLRT). Lower values for AIC, BIC, and aBIC indicate superior model fit. Entropy values closer to 1 indicate better classification precision. LMR and BLRT tests with *p* < 0.05 suggest that the k-class model fits better than the k-1 class model. The BLRT used 1000 bootstrap resamples. The optimal model was selected based on a combination of statistical fit indices, classification quality (entropy), and clinical interpretability of the profiles. As shown in Table 2, the 3-profile solution was selected because it demonstrated the highest entropy (0.912), a significant LMR test (*p* = 0.0012), and clinically meaningful distinctions among subgroups, whereas the 4-profile solution had a non-significant LMR test (*p* = 0.0625).

Prior to conducting multinomial logistic regression, the study examined variance inflation factors (VIF) to assess potential multicollinearity among independent variables. All VIF values were below 2.5, indicating that multicollinearity was not a concern.

To account for classification uncertainty in the subsequent regression analysis, this study employed a three-step approach as implemented in Mplus [43]. This method incorporates classification error probabilities into the regression model, reducing bias in parameter estimates. The results of the three-step approach were consistent with the standard multinomial logistic regression conducted in SPSS.

Group differences in demographic characteristics (age, BMI, education level, etc.), weight stigma (PWSQ, WBIS), and eating behaviors (emotional, external, restrained eating) across the identified PPD profiles were first examined using Welch’s ANOVA (for continuous variables) and Chi-square tests (for categorical variables). Variables with significant group differences (*P* < 0.05) were included in multinomial logistic regression (conducted in SPSS 29.0) to explore factors associated with PPD profile membership.

### Ethical considerations

The study received ethical approval from the Ethics Committee of Xiangya School of Nursing, Central South University (Approval No. E2021108). Before data collection, participants were fully informed about the study’s purpose, procedures, risks, and benefits. Both verbal and written informed consent were obtained from all participants. All collected data were encrypted and stored securely, with access restricted to the research team only. The study was conducted in accordance with the Declaration of Helsinki.

## Result

### Demographic characteristics

A total of 507 valid questionnaires were included in the final analysis. The mean age of the participants was 30.92 years (SD = 4.67), with the majority residing in urban areas (68.2%). Most participants held a university degree (70.6%). The average BMI among the participants was 22.94 (SD = 3.00). (See Table 1).

**Table 1:** Demographic characteristics of postpartum women

Variables	Categories	*N* (%)	Mean (SD)
Age			30.92 (4.67)
BMI			22.94 (3.00)
PPWR			4.72 (6.30)
Residence	Rural	73 (14.4)	
	Urban	346 (68.2)	
	Suburb	88 (17.4)	
Education level	High school and below	71 (14.0)	
	University	358 (70.6)	
	Postgraduate and above	78 (15.4)	
Monthly income (RMB)	< 3000	44 (8.7)	
	3000 ~ 5000	119 (23.5)	
	>5000	344 (67.9)	
Number of births	One	305 (60.2)	
	Two	184 (36.3)	
	Three and above	18 (3.6)	
Type of childbirth	Natural delivery	304 (60.2)	
	Cesarean surgery	203 (40.0)	

Note: *BMI=* Body mass index, *PPWR=* Postpartum weight retention

### Results of latent profile analysis

In this study, ten items of EPDS were used as exogenous variables to establish a latent profile model, and 1–4 potential categories were explored for model fitting. As shown in Table 2, AIC, BIC, and aBIC values decreased progressively from the 1-profile to the 4-profile solutions. The 3-profile model demonstrated the highest entropy (0.912), indicating excellent classification accuracy. While the 4-profile model showed lower AIC and BIC values, the LMR test was non-significant (*P* = 0.0625), suggesting that the 4-profile solution did not provide a statistically significant improvement over the 3-profile model. Additionally, the 4-profile model included a very small subgroup (17%) that was not clinically distinguishable from adjacent profiles. Therefore, the 3-profile solution was selected based on statistical fit, parsimony, and clinical interpretability. The attribution probability matrix for the three potential profiles is presented in Table 3. The average probability of each category being attributed to its corresponding potential profile ranged from 94.1% to 98.3% (all > 90%), suggesting reasonable results for the three potential profiles in this study.

**Table 2:** Indicators for each latent profile of depression in postpartum women

Model	Likelihood	AIC	BIC		aBIC	*P*LMR	*P*BLRT	Entropy	Proportion
Class1	-6054.183	12148.365		12232.935	12169.453				
Class 2	-5209.489	10480.977	10612.061		10513.663	0.0000	0.0000	0.895	0.58/0.42
Class 3	-4914.274	9912.548	10090.146		9956.833	0.0012	0.0000	0.912	0.45/0.42/0.13
Class 4	-4809.717	9725.434	9949.545		9781.317	0.0625	0.0000	0.860	0.37/0.33/0.13/0.17

Note: Bold entries reflect the selected model. Class refers to the number of potential categories. Class 1 to Class 4 means that it is divided into 1–4 potential categories. Through model comparison, the optimal number of categories is determined

**Table 3:** Indicators for each latent profile of depression in postpartum women

Category	Profile 1	Profile 2	Profile 3
Profile 1	0.968	0.032	0.000
Profile 2	0.049	0.941	0.010
Profile 3	0.000	0.017	0.983

Note: Category means that it is divided into 3 potential categories

### Naming of latent profile

**Fig. 1:**
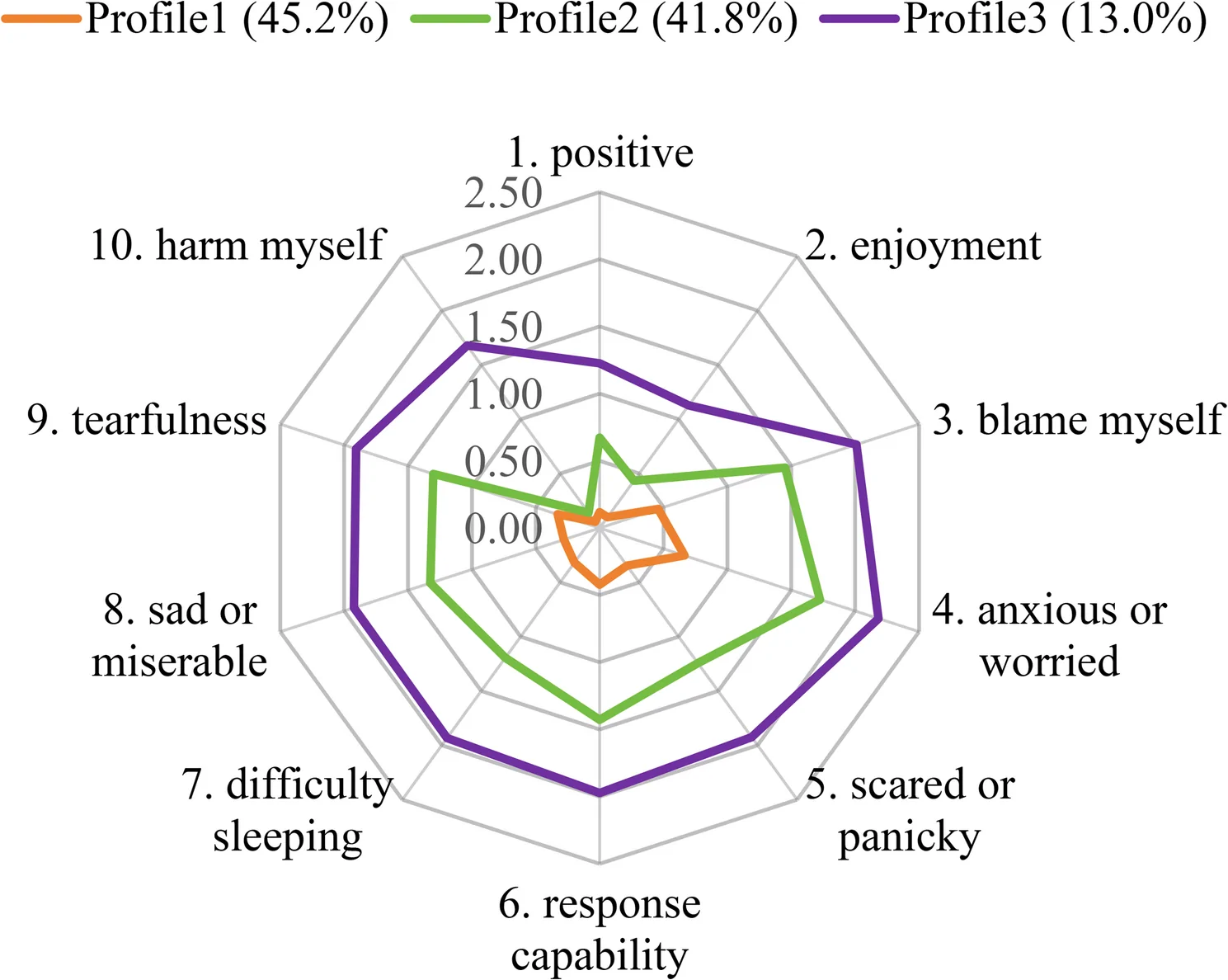
Latent profile model of PPD among postpartum women. Note: Numbers 1–10 in this figure are the number of EPDS scale items

Figure 1 reflected the mean values of the three profiles of PPD levels in terms of the scores of each item. Profile 1, labeled ‘low-level PPD’, was the largest category, representing approximately 45.2% of the subjects (*n* = 229) with a low level of PPD. Profile 3, named ‘high-level PPD’, comprised the smallest percentage of subjects at 13.0% (*n* = 66) and exhibited the highest level of PPD. Profile 2, labeled ‘middle-level PPD’, included 41.8% of the subjects (*n* = 212) and was characterized by a moderate level of PPD.

### Inter-profile characteristic differences

The results of the univariate analysis indicated significant differences in BMI, PWSQ, WBIS, restrained eating, emotional eating, and external eating (*P* < 0.05). (See Table 4).

**Table 4:** Inter-profile characteristic differences (N, %)

Variable	Profile 1	Profile 2	Profile 3	χ ^2^/F	*P*
Residence				4.471	0.346
Rural	30 (13.1)	29 (13.7)	14 (21.2)		
Urban	157 (68.6)	144 (67.8)	45 (68.2)		
Suburb	42 (18.3)	39 (18.4)	7 (10.6)		
Education level				7.161	0.128
High school and below	26 (11.4)	30 (14.2)	15 (22.7)		
University	163 (71.2)	150 (70.8)	45 (68.2)		
Postgraduate and above	40 (17.5)	32 (15.1)	6 (9.1)		
Monthly income (RMB)				1.049	0.902
< 3000	17 (7.4)	20 (9.4)	7 (10.6)		
3000 ~ 5000	53 (23.1)	51 (24.1)	15 (22.7)		
>5000	159 (69.4)	141 (66.5)	44 (66.7)		
Number of births				4.797	0.309
One	130 (56.8)	138 (65.1)	37 (56.1)		
Two	91 (39.7)	68 (32.1)	25 (37.9)		
Three and above	8 (3.5)	6 (2.8)	4 (6.1)		
Delivery method				1.306	0.521
Natural delivery	136 (59.4)	132 (62.3)	36 (54.5)		
Cesarean surgery	93 (40.6)	80 (37.7)	30 (45.5)		
Medical Payment				0.678	0.712
Out-of-Pocket	25 (10.9)	28 (13.2)	9 (13.6)		
Health Insurance	204 (89.1)	184 (86.8)	57 (86.4)		
Age (mean ± SD)	30.96 ± 4.22	31.17 ± 5.04	29.95 ± 4.84	1.729	0.178
BMI (mean ± SD)	23.12 ± 2.98	22.56 ± 2.88	23.58 ± 3.35^b^	3.606	0.028*
PWSQ (mean ± SD)	0.15 ± 0.58	0.53 ± 1.29^a^	1.29 ± 2.04^ab^	24.487	<0.001*
WBIS (mean ± SD)	23.17 ± 8.50	25.79 ± 8.65^a^	32.67 ± 9.96^ab^	30.285	<0.001*
PPWR (mean ± SD)	4.95 ± 6.02	4.38 ± 6.48	4.98 ± 6.75	0.502	0.606
Restrained eating (mean ± SD)	22.05 ± 8.84	25.37 ± 8.35^a^	26.88 ± 8.06^ab^	12.441	<0.001*
Emotional eating (mean ± SD)	23.69 ± 10.19	27.94 ± 10.43^a^	32.48 ± 11.58^ab^	20.960	<0.001*
External eating (mean ± SD)	28.03 ± 8.19	31.15 ± 5.68^a^	32.65 ± 5.59^a^	17.046	<0.001*

The variance analysis was the Welch method. Variables with homogeneous variance and the LSD test were used for post-hoc multiple comparisons. Variables with unequal variance were compared by Dunnett’s T3 method

*Statistically significant

^a^ indicates that compared with Profile 1, *P* < 0.05, ^b^ indicates that compared with Profile 2, *P* < 0.05

### Multinomial logistic regression of depression profiles

In this study, PPD profiles were used as the dependent variable, while BMI, PWSQ, WBIS, restrained eating, emotional eating, and external eating (*P* < 0.05) served as independent variables. To avoid omitting important variables, sociodemographic factors with a univariate analysis result of *P* < 0.20 (age, education level) were also included as independent variables for analysis.

**Fig. 2:**
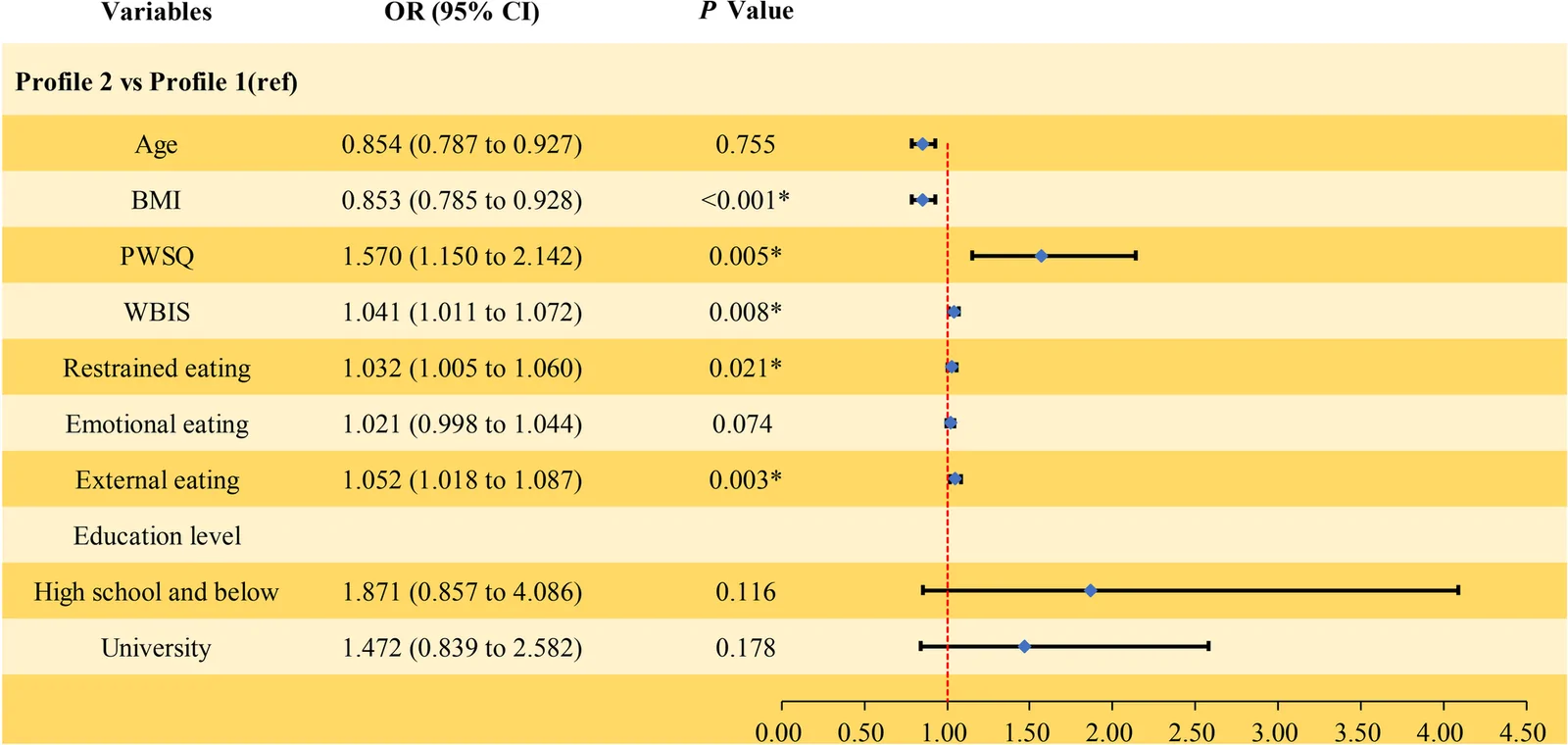
Multinomial logistic regression of depression profiles: Profile 2 vs. Profile 1(ref)

**Fig. 3:**
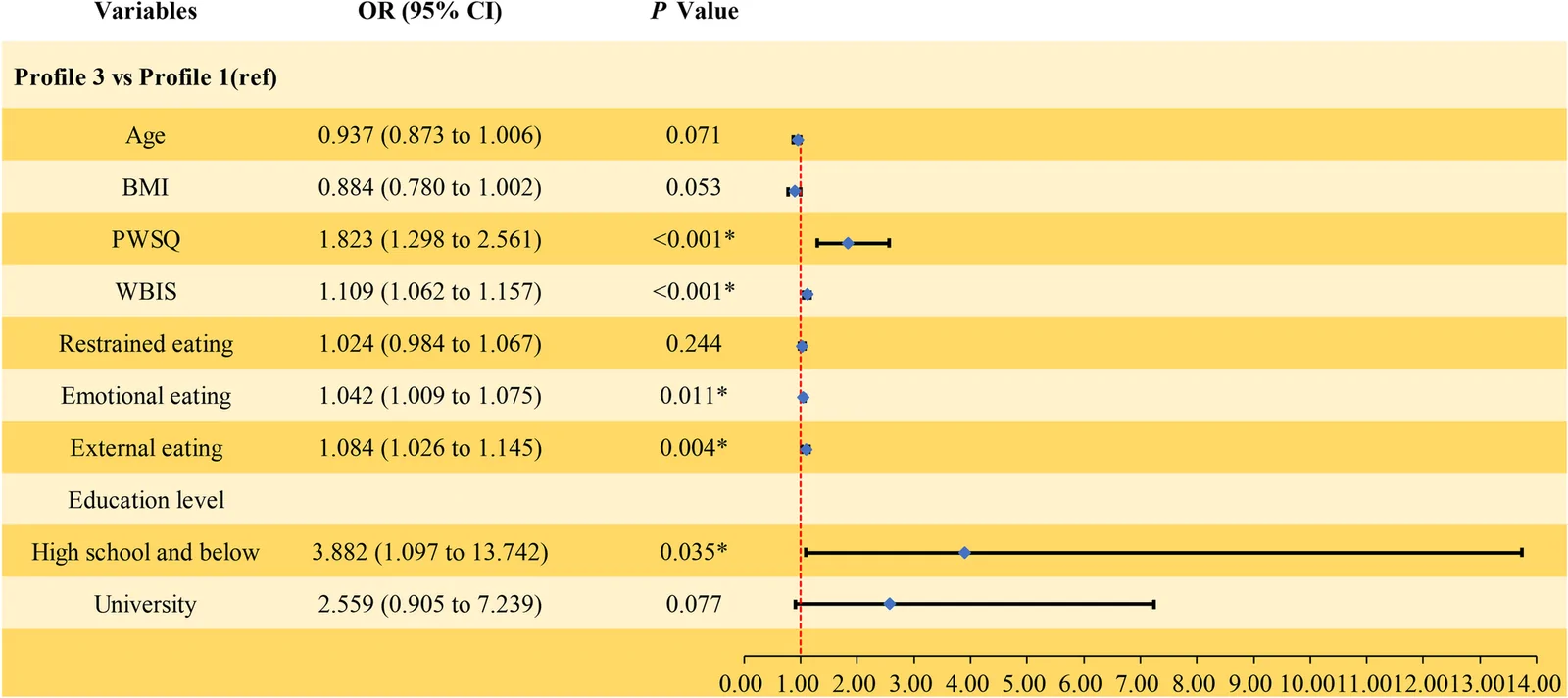
Multinomial logistic regression of depression profiles: Profile 3 vs. Profile 1(ref)

**Fig. 4:**
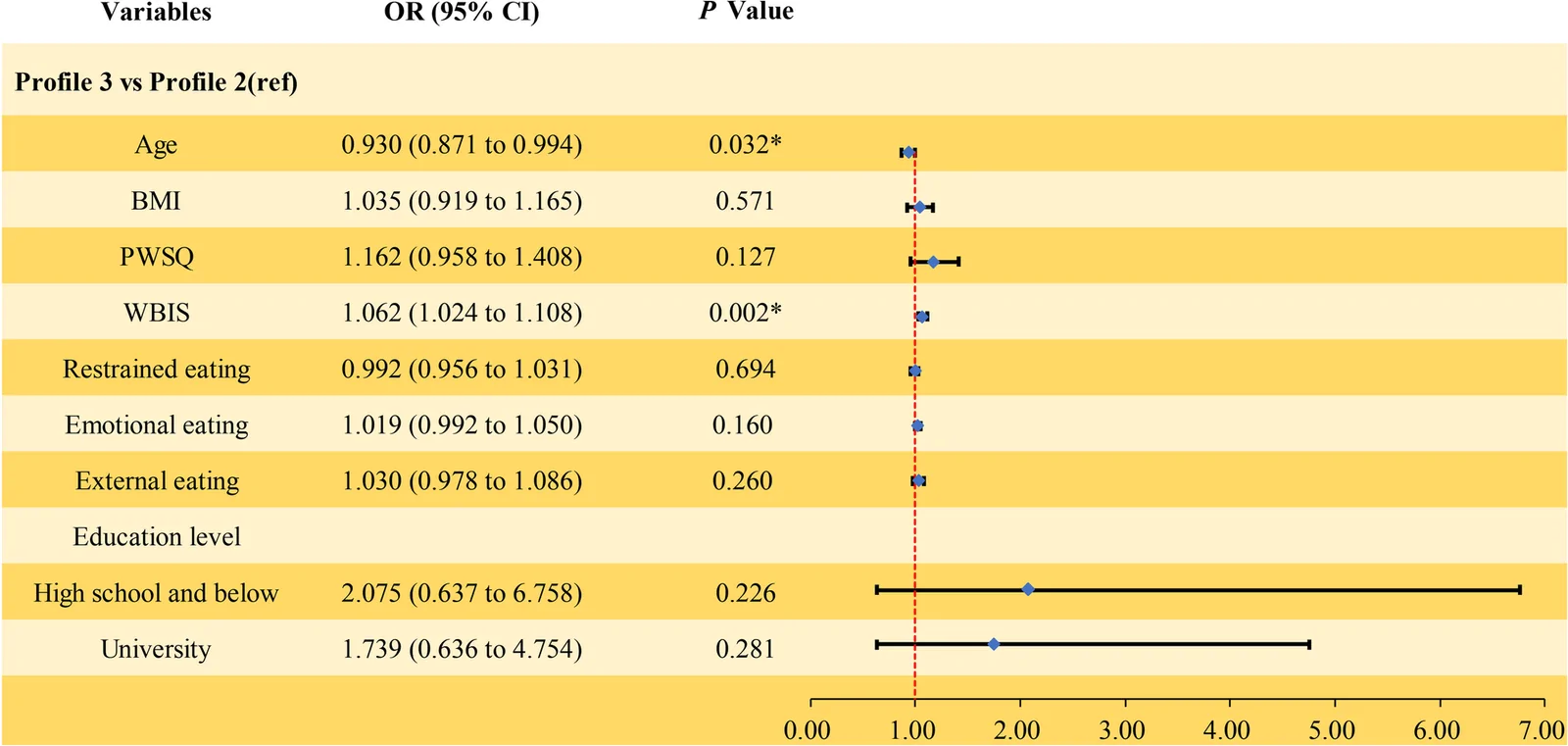
Multinomial logistic regression of depression profiles: Profile 3 vs. Profile 2(ref)

As detailed in Figs. 2, 3 and 4, age, BMI, PWSQ, WBIS, restrained eating, emotional eating, external eating, and high school and below education level were significantly associated with the PPD profiles. Specifically, compared to Profile 1, membership in Profile 2 was significantly associated with higher PWSQ (OR = 1.570, 95%CI = 1.150–2.142), WBIS (OR = 1.041, 95%CI = 1.011–1.072), restrained eating (OR = 1.032, 95%CI = 1.005–1.060), external eating (OR = 1.052, 95%CI = 1.018–1.087). Higher BMI was associated with lower odds of belonging to Profile 2 compared to Profile 1 (OR = 0.853, 95%CI = 0.785–0.928). When comparing Profile 3 to Profile 1, membership in Profile 3 was associated with higher PWSQ (OR = 1.823, 95%CI = 1.298–2.561), WBIS (OR = 1.109, 95%CI = 1.062–1.157), emotional eating (OR = 1.042, 95%CI = 1.009–1.075), and external eating (OR = 1.084, 95%CI = 1.026–1.145), and lower education level (high school or below) (OR = 3.882, 95%CI = 1.097–13.742). Additionally, compared to Profile 2, membership in Profile 3 was significantly associated with higher WBIS (OR = 1.062, 95%CI = 1.024–1.108), while younger age was associated with higher odds of belonging to Profile 3 compared to Profile 2 (OR = 0.930, 95%CI = 0.871–0.994).

## Discussion

To our knowledge, this study is among the first to apply LPA to investigate PPD symptom profiles among Chinese postpartum women, offering a person-centered framework for identifying subgroups with distinct associated characteristics. LPA revealed three distinct profiles, which were primarily distinguished by symptom severity: ‘low-level PPD’ (Profile 1, 45.2%), ‘middle-level PPD’ (Profile 2, 41.8%), and ‘high-level PPD’ (Profile 3, 13.0%). This finding highlights the heterogeneity of PPD presentations and provides a basis for exploring correlates that vary across severity levels.

Profile 1, representing the largest group, exhibited the lowest mean scores across all EPDS items, ranging from 0.06 to 0.66, indicative of generally low levels of depressive symptoms. However, even within this ostensibly low-risk group, certain items, such as item 4 (“I have been anxious or worried for no good reason”) and item 3 (“I have blamed myself unnecessarily when things went wrong”), showed relatively higher mean scores. These findings suggest specific areas that may warrant attention. Anxiety and self-blame, as reflected in these items, have been well-documented as factors associated with the development of PPD [44, 45]. Intervention strategies for the low-level PPD group could consider addressing issues related to anxiety and self-blame, as these may help mitigate the worsening of depressive symptoms within this largest low-risk population. In addition, these findings further highlight the strengths of LPA in characterizing different subgroups based on individual responses [28], thus enabling more precise and targeted interventions for PPD.

The middle-level PPD group (Profile 2) exhibited higher mean scores across all EPDS items than the low-level PPD group (Profile 1). However, the similarity in scores for item 10 (self-harm) between Profiles 2 and 1-both approaching zero-suggests that self-harm may not be a distinguishing feature in these subgroups. In contrast, the high-level PPD group (Profile 3) scored consistently higher on all items, especially item 10. This significant difference underscores the critical need to pay special attention to item 10. Cox [46], the original developer of the EPDS, along with the Chinese Center for Disease Control and Prevention [47], have both emphasized that any non-zero score on item 10 of the EPDS should trigger a comprehensive clinical assessment to ensure timely identification and intervention for potential suicide risk.

However, the validity and clinical utility of item 10 have been subject to ongoing debate. Previous research suggests that item 10, designed to assess suicidal ideation, is often misinterpreted by respondents as referring to accidental or unintended harm, potentially leading to an overestimation of suicide risk [48, 49]. This misinterpretation can result in unnecessary resource allocation, as any positive response to item 10 typically triggers comprehensive psychiatric evaluations. Furthermore, a study conducted in Japan [50] demonstrated that the EPDS-9, an abbreviated version excluding the self-harm item, performed comparably to the full EPDS in screening for perinatal depression. These findings suggest that universal screening for self-harm may not be necessary for all perinatal women and that more selective screening among high-risk individuals might be a more effective approach. Meanwhile, in this study, the similarity in scores on item 10 between Profile 1 and Profile 2 suggests that self-harm tendencies are not a key distinguishing characteristic between these two subgroups. Further research is required to clarify these issues.

The three-profile solution identified in this study (low, middle, high) aligns with several international studies that have reported severity-based profiles of postpartum depression. The landmark study by the Postpartum Depression: Action Towards Causes and Treatment (PACT) Consortium, which aggregated data from 19 institutions across seven countries (*N* = 17,912), applied latent class analysis to EPDS items and identified three distinct classes characterized primarily by symptom severity: a mild class (mean EPDS = 3.3), a moderate class (mean EPDS = 12.3), and a severe class (mean EPDS = 20.3) [51]. This three-class solution has been widely replicated; a systematic review by Santos and colleagues concluded that most studies examining PPD heterogeneity identify either three or four distinct trajectories, varying primarily in severity (low, medium, high) [52]. More recently, a comprehensive systematic review by Waqas et al. confirmed that three-class solutions are the most commonly identified patterns across studies of perinatal depression symptom profiles [53].

However, some studies have identified qualitatively distinct profiles beyond severity. For instance, a study by Carona and colleagues examined mental health profiles among 1,013 women at high-risk for PPD and identified three profiles: “Good Mental Health” (low psychopathology, high positive mental health), “Moderate Mental Health” (moderate depression/anxiety, moderate positive mental health), and “Impaired Mental Health” (high depression/anxiety, low positive mental health) [54]. These profiles were distinguished not only by symptom severity but also by levels of positive mental health, emotion regulation, psychological flexibility, and maternal self-efficacy. The discrepancy between our findings (severity-based profiles) and studies reporting qualitatively distinct profiles may reflect differences in measurement instruments, sample characteristics, or the populations under study. Our sample comprised general postpartum women, whereas Carona et al. specifically recruited women at high-risk for PPD, which may have enabled the identification of more nuanced qualitative distinctions. Additionally, our use of EPDS items as the sole basis for profile identification may be more sensitive to severity than to symptom-specific patterns, as the EPDS was designed as a screening tool rather than a comprehensive measure of depressive symptom heterogeneity.

The second objective of this study was to explore the correlates of PPD among postpartum women. The results revealed that perceived weight stigma, weight bias internalization, restrained eating, emotional eating, external eating, age, BMI, and education level were all significantly associated with PPD. Compared to low-level PPD, perceived weight stigma, and weight bias internalization emerged as significant associated factors, consistent with existing literature [55, 56]. However, the current study uncovers nuanced differences in these associations. Specifically, no significant relationship between perceived weight stigma and PPD was observed when comparing women with high-level PPD to those with middle-level PPD. One possible interpretation is that while perceived weight stigma is associated with PPD, its association may diminish or plateau once depressive symptoms reach a more severe threshold. In contrast, weight bias internalization remained robustly associated with PPD across all levels, suggesting that weight stigma may be most consequential when internalized. Internalized weight stigma occurs when postpartum women accept and endorse weight-based stereotypes and beliefs as true about themselves [57]. Previous research has also shown that internalized weight stigma mediates the association between weight stigma and psychological distress [58]. Specifically, weight stigma experiences are positively associated with weight bias internalization, which in turn is associated with psychological distress. The distinct characteristics of these two forms of stigma contribute to the nuanced findings in the present study: perceived weight stigma is often situational and externally influenced, whereas weight stigma internalization represents a more enduring and deeply ingrained psychological process. Based on these findings, interventions aimed at reducing PPD might benefit from addressing not only perceived weight stigma but also, and perhaps more importantly, weight stigma internalization. Specifically, interventions targeting the weight stigma internalization—a psychological mechanism involving self-perception and self-blame—may have a more pronounced positive impact on women experiencing severe PPD.

Eating behaviors also emerged as complex correlates of PPD in this study. We examined the relationships between three eating behaviors—restrained eating, emotional eating, and external eating—and PPD. The findings revealed that, compared to the low-level PPD group, restrained and external eating were positively associated with middle-level PPD, while all three eating behaviors were positively associated with high-level PPD, consistent with previous research [59, 60]. A longitudinal study conducted in the UK demonstrated that both current and pre-existing eating disorders, including restrained eating, were strongly associated with higher levels of PPD and anxiety [59]. Similarly, Paans, Bot [60] found positive associations between external and emotional eating and depressive symptoms. These results highlight the role of maladaptive eating patterns in the psychological well-being of postpartum women and suggest that interventions addressing these behaviors may be relevant to PPD risk.

This study further uncovered differential associations between various eating behaviors and distinct PPD profiles. First, when comparing high-level PPD (Profile 3) with low-level PPD (Profile 1), restrained eating did not emerge as a significant factor. One possible explanation is that greater cognitive impairment often associated with severe depression may hinder the ability to maintain restrained eating behaviors [61]. Second, emotional eating was not significant when comparing middle-level PPD (Profile 2) to low-level PPD (Profile 1), a finding that is consistent with emotion regulation theory. This theory posits that individuals experiencing high levels of stress or depression tend to seek immediate emotional relief through the consumption of high-sugar, high-fat ‘comfort foods’ [62]. At lower levels of PPD, however, individuals may retain some capacity for self-regulation, which could reduce the impact of emotional eating on depressive symptoms. Finally, no significant association was observed between any of the three eating behaviors and PPD when comparing high-level to middle-level PPD. This finding may suggest a degree of continuity between these two profiles, where differences in eating behaviors are insufficient to distinguish between them. Alternatively, in more severe stages of PPD, changes in eating behaviors may no longer be a core feature, with deeper psychological factors contributing to the progression of PPD. This study provides insights into the relationship between PPD and eating behaviors, emphasizing the potential value of personalized dietary interventions tailored to various subgroups. Future research should investigate the potential pathways through which these mechanisms function, incorporating multidimensional factors to facilitate the development of more targeted and effective interventions.

In terms of demographic factors, this study found that younger age was associated with a higher likelihood of belonging to the high-level PPD profile compared to the middle-level PPD profile. Younger mothers have been shown to exhibit lower PPD literacy [63], including limited knowledge of self-care activities and appropriate help-seeking attitudes, which may contribute to higher PPD severity. Additionally, comparisons between high and low levels of PPD revealed that women with lower educational attainment (high school or below) were more likely to belong to the high-level PPD profile compared to those with higher education (graduate school and above). This finding is consistent with previous research [64, 65]. These findings suggest that cognitive resources may play an important role in addressing postpartum challenges. Lower educational levels may be associated with reduced access to mental health services, coping strategies, or awareness of available support systems. Additional support could be considered for younger and less educated postpartum women, particularly in the areas of early identification and prevention.

Regarding BMI, we found that higher BMI was associated with a lower likelihood of belonging to the low-level PPD profile (Profile 1) compared to the middle-level PPD profile (Profile 2). One possible interpretation, which warrants further investigation, relates to the cultural practice of ‘sitting the month’ (zuò yuèzi), during which weight gain is traditionally viewed as a sign of adequate nourishment and successful recovery [66]. However, as we did not directly assess adherence to or attitudes toward this practice, this interpretation remains speculative. Future studies should include direct measures of cultural practices to examine their potential role in shaping the relationship between BMI and PPD. Additionally, the concept of familial responsibility holds significant weight in Chinese culture [67], and the inability to fulfill these roles—particularly in terms of effective parenting—may lead to heightened feelings of guilt [68]. Thus, it is possible that a higher postpartum BMI is associated with better perceived physical health, improved breastfeeding outcomes, or enhanced confidence in fulfilling familial responsibilities, which in turn may be associated with lower PPD severity. These findings emphasize the need to consider cultural context when developing interventions for postpartum women.

However, BMI was not identified as a significant factor in comparisons involving higher levels of PPD (Profile 3 vs. Profile 1, and Profile 3 vs. Profile 2), suggesting that there may be a threshold beyond which its association with PPD is limited. This finding suggests that the potential influence of any single cultural factor may be diminished when more severe PPD symptoms are present, highlighting the complex interplay of factors that may contribute to the progression of postpartum depression.

## Limitations

This study has several limitations that should be considered when interpreting the findings. First, the cross-sectional design precludes any conclusions about causality or temporal ordering among the variables. The associations identified should be interpreted as correlational rather than causal, and longitudinal studies are needed to clarify the directionality of these relationships. Second, all measures were self-reported, raising the possibility of common method bias. Although all scales demonstrated acceptable internal consistency, future studies would benefit from incorporating clinical interviews or objective measures to complement self-report data. The EPDS is a screening tool rather than a diagnostic instrument, and the three profiles identified were primarily distinguished by symptom severity rather than qualitatively distinct patterns, suggesting that the EPDS items may capture a unidimensional severity continuum. Third, convenience sampling and the demographic composition of the sample (predominantly urban residents [68.2%] and women with university education [70.6%]) limit the generalizability of the findings to postpartum women in rural areas or with lower socioeconomic status. Future studies should aim to increase the geographic and demographic diversity of the sample. Fourth, several potentially important variables were not assessed, including social support, sleep quality, marital satisfaction, and pre-existing mental health conditions. These factors have been shown to be associated with PPD in previous research and may confound the relationships observed in this study. The study also did not stratify by postpartum timing (one week to one year), which may obscure stage-specific patterns across the postpartum period. Fifth, the Chinese versions of the PWSQ, WBIS, and DEBQ have been primarily validated in non-postpartum populations. Although these scales demonstrated acceptable internal consistency in the current sample and have been used in a previous study with the same dataset, further validation of their psychometric properties specifically in Chinese postpartum populations is warranted.

## Conclusions

This study identified three distinct profiles of postpartum depression among Chinese women, characterized primarily by symptom severity: low-level, middle-level, and high-level. Perceived weight stigma, weight bias internalization, and maladaptive eating behaviors emerged as key factors associated with profile membership, underscoring the relevance of these psychosocial variables alongside sociodemographic characteristics (e.g., age, BMI, education level) in postpartum care. By applying a person-centered approach, these findings offer a nuanced understanding of postpartum depression heterogeneity and provide preliminary evidence to guide the development of tailored intervention strategies. Addressing profile-specific characteristics—particularly weight-related stigma and eating behaviors—may contribute to more targeted prevention and treatment, ultimately advancing the precision of postpartum mental healthcare.

## Data Availability

The data that support the findings of this study are available on request from the corresponding author. The data are not publicly available due to privacy or ethical restrictions.

## References

[1] Stuart-Parrigon K, Stuart S. Perinatal depression: an update and overview. Curr Psychiatry Rep. 2014;16(9):468. 10.1007/s11920-014-0468-6 PMC4920261 25034859

[2] Wang Z, et al. Mapping global prevalence of depression among postpartum women. Transl Psychiatry. 2021;11(1):543. 10.1038/s41398-021-01663-6 PMC8528847 34671011

[3] Roddy Mitchell A, et al. Prevalence of Perinatal Depression in Low- and Middle-Income Countries: A Systematic Review and Meta-analysis. JAMA Psychiatry. 2023;80(5):425–31. 10.1001/jamapsychiatry.2023.0069 PMC9996459 36884232

[4] Nisar A, et al. Prevalence of perinatal depression and its determinants in Mainland China: A systematic review and meta-analysis. J Affect Disord. 2020;277:1022–37. 10.1016/j.jad.2020.07.046 33065811

[5] Hurwitz E, et al. Harnessing Consumer Wearable Digital Biomarkers for Individualized Recognition of Postpartum Depression Using the All of Us Research Program Data Set: Cross-Sectional Study. JMIR Mhealth Uhealth. 2024;12:e54622. 10.2196/54622 PMC11099816 38696234

[6] Sangsawang N, Sangsawang B. Postpartum depression, social support and maternal self-efficacy between adolescent and adult mothers during the COVID-19 pandemic: A comparative cross-sectional study. J Adv Nurs. 2023;79(1):113–24. 10.1111/jan.15445 36117329

[7] Lawrence PJ, et al. Intrusive thoughts and images of intentional harm to infants in the context of maternal postnatal depression, anxiety, and OCD. Br J Gen Pract. 2017;67(661):376–7. 10.3399/bjgp17X692105 PMC5519121 28751351

[8] Shi P, et al. Maternal depression and suicide at immediate prenatal and early postpartum periods and psychosocial risk factors. Psychiatry Res. 2018;261:298–306. 10.1016/j.psychres.2017.12.085 29331710

[9] Chorbadjian TN, et al. Maternal Depressive Symptoms and Developmental Delay at Age 2: A Diverse Population-Based Longitudinal Study. Matern Child Health J. 2020;24(10):1267–77. 10.1007/s10995-020-02990-8 32780269

[10] Ye Z, et al. Gender of infant and risk of postpartum depression: a meta-analysis based on cohort and case-control studies. J Matern Fetal Neonatal Med. 2022;35(13):2581–90. 10.1080/14767058.2020.1786809 32635787

[11] Li C. A qualitative study of risk factors and intervention strategies for postpartum depression based on the family support perspective. 2022.

[12] McNab SE, et al. The silent burden: a landscape analysis of common perinatal mental disorders in low- and middle-income countries. BMC Pregnancy Childbirth. 2022;22(1):342. 10.1186/s12884-022-04589-z PMC9019797 35443652

[13] NHSC. Exploration of Special Service Work Plan for Depression Prevention and Treatment. 2020.

[14] Bliddal M, et al. Mental disorders in motherhood according to prepregnancy BMI and pregnancy-related weight changes–A Danish cohort study. J Affect Disord. 2015;183:322–9. 10.1016/j.jad.2015.04.053 26047960

[15] Emerson JA et al. Maternal mental health symptoms are positively related to emotional and restrained eating attitudes in a statewide sample of mothers participating in a supplemental nutrition program for women, infants and young children. Matern Child Nutr, 2017. 13(1). 10.1111/mcn.12247 PMC6866000 26898604

[16] Nippert KE, et al. The Media as a Source of Weight Stigma for Pregnant and Postpartum Women. Obes (Silver Spring). 2021;29(1):226–32. 10.1002/oby.23032 33215866

[17] Puhl R, Himmelstein, Pearl RA-O. Weight stigma as a psychosocial contributor to obesity*.* (1935-990X (Electronic)). 10.1037/amp0000538 32053000

[18] Sun M, Peng J, Lommel L. Prevalence and Correlates of Weight Stigma among Postpartum Women in China. LID – 10.3390/ijerph192214692IF: NA NA NA. LID – 14692. (1660–4601 (Electronic)). 10.3390/ijerph192214692 PMC9690326 36429415

[19] Pearl RA-O, Puhl RM. Weight bias internalization and health: a systematic review. Obes reviews: official J Int Association Study Obes. 2018;19(8):1141–63. 10.1111/obr.12701 PMC6103811 29788533

[20] Peng J, et al. Perceived weight stigma and disordered eating behaviors among postpartum women: The mediating role of weight bias internalization and postpartum depression. Int J Nurs Stud Adv. 2025;8:100306. 10.1016/j.ijnsa.2025.100306 PMC11867231 40018052

[21] Tsai SS, Wang HH. Role changes in primiparous women during ‘doing the month’ period. Midwifery. 2019;74:6–13. 10.1016/j.midw.2019.03.007 30908975

[22] Wong J, Fisher J. The role of traditional confinement practices in determining postpartum depression in women in Chinese cultures: a systematic review of the English language evidence. J Affect Disord. 2009;116(3):161–9. 10.1016/j.jad.2008.11.002 19135261

[23] Sun M, Peng J, Lommel L. Prevalence and Correlates of Weight Stigma among Postpartum Women in China. Int J Environ Res Public Health, 2022. 19(22). 10.3390/ijerph192214692 PMC9690326 36429415

[24] Van Strien T, W.M. Schippers Gm Fau - Cox, and, Cox WM. On the relationship between emotional and external eating behavior. (0306–4603 (Print)). 10.1016/0306-4603(95)00018-8 8712056

[25] Al-Abri K, Edge D, Armitage CJ. Prospective analysis of factors associated with perinatal depression. Midwifery. 2024;128:103871. 10.1016/j.midw.2023.103871 37967499

[26] Su X, et al. Influencing factors and risk prediction modeling of maternal postpartum depression: a cross-sectional study in Chinese puerperal women of sitting the month. Front Psychiatry. 2023;14:1252789. 10.3389/fpsyt.2023.1252789 PMC10539603 37779623

[27] Aguinis H, Gottfredson RK. Best-practice recommendations for estimating interaction effects using moderated multiple regression. J Organizational Behav. 2010;31(6):776–86.

[28] Tan. Y. Application of LPA in Mental Health Research, in Advances in Social Sciences. 2024;383–389.

[29] Branquinho MA-O, Canavarro MA-O, Fonseca AA-O. Profiles of women with postpartum depressive symptoms: A latent profile analysis using the Edinburgh Postnatal Depression Scale factors. J Clin Psychol. 2022;78(7):1500–15. 10.1002/jclp.23320 35098527

[30] Liu H, et al. Categories, symptom characteristics and factors influencing depression and anxiety in pregnant women: latent profile analysis and network analysis. Psychiatry Res. 2026;356:116912. 10.1016/j.psychres.2025.116912 41422579

[31] Dewidar O, et al. Improving the Reporting on Health Equity in Observational Research (STROBE-Equity): Extension Checklist and Elaboration. BMJ (Clinical Res ed). 2025;390:e083882. 10.1136/bmj-2024-083882 PMC12406149 40903054

[32] Spurk D et al. Latent profile analysis: A review and how to guide of its application within vocational behavior research. J Vocat Behav, 2020. 120.

[33] Crompvoets PA-O, et al. Perceived weight stigma in healthcare settings among adults living with obesity: A cross-sectional investigation of the relationship with patient characteristics and person-centred care. Health expectations: Int J public participation health care health policy. 2024;27(1):e13954. 10.1111/hex.13954 PMC10790109 39102661

[34] Lin CY, et al. Mediated effects of eating disturbances in the association of perceived weight stigma and emotional distress. Eat Weight Disord. 2020;25(2):509–18. 10.1007/s40519-019-00641-8 30697663

[35] Peng J et al. Perceived weight stigma and disordered eating behaviors among postpartum women: The mediating role of weight bias internalization and postpartum depression. (2666-142X (Electronic)). 10.1016/j.ijnsa.2025.100306 PMC11867231 40018052

[36] Durso LE, Latner JD. Understanding self-directed stigma: development of the weight bias internalization scale. Obes (Silver Spring). 2008;16(Suppl 2):S80–6. 10.1038/oby.2008.448 18978768

[37] Ciupitu-Plath C, Wiegand S, Babitsch B. The Weight Bias Internalization Scale for Youth: Validation of a Specific Tool for Assessing Internalized Weight Bias Among Treatment-Seeking German Adolescents With Overweight. J Pediatr Psychol. 2018;43(1):40–51. 10.1093/jpepsy/jsx079 28482018

[38] Pakpour AH, et al. Psychometric properties and measurement invariance of the Weight Self-Stigma Questionnaire and Weight Bias Internalization Scale in children and adolescents. Int J Clin Health Psychol. 2019;19(2):150–9. 10.1016/j.ijchp.2019.03.001 PMC6517648 31193103

[39] Cox JL, Holden JM, Sagovsky R. Detection of postnatal depression. Development of the 10-item Edinburgh Postnatal Depression Scale. Br J Psychiatry. 1987;150:782–6. 10.1192/bjp.150.6.782 3651732

[40] Lee DT, et al. Detecting postnatal depression in Chinese women. Validation of the Chinese version of the Edinburgh Postnatal Depression Scale. Br J Psychiatry. 1998;172:433–7. 10.1192/bjp.172.5.433 9747407

[41] van Strien T, Frijters JER, Bergers GPA, Defares PB. The Dutch Eating Behavior Questionnaire (DEBQ) for assessment of restrained, emotional, and external eating behavior. Int J Eat Disord. 1986;5(2):295–315.

[42] Zhang J et al. Emotional Eating in Pregnant Women during the COVID-19 Pandemic and Its Association with Dietary Intake and Gestational Weight Gain. LID – 10.3390/nu12082250. LID – 2250. (2072–6643 (Electronic)). 10.3390/nu12082250 PMC7468999 32731389

[43] Vermunt JK. Latent Class Modeling with Covariates: Two Improved Three-Step Approaches. Political Anal. 2017;18(4):450–69.

[44] Stewart DE, Vigod SN. Postpartum Depression: Pathophysiology, Treatment, and Emerging Therapeutics. Annu Rev Med. 2019;70:183–96. 10.1146/annurev-med-041217-011106 30691372

[45] Gutiérrez-Zotes A, et al. Coping strategies for postpartum depression: a multi-centric study of 1626 women. Arch Womens Ment Health. 2016;19(3):455–61. 10.1007/s00737-015-0581-5 26399872

[46] Cox J. Thirty years with the Edinburgh Postnatal Depression Scale: voices from the past and recommendations for the future. Br J Psychiatry. 2019;214(3):127–9. 10.1192/bjp.2018.245 30774059

[47] China Disease Control Center. Determination of Postpartum Depression. 2021 [cited 2024 August 20]; Available from: https://www.chinawch.org.cn/jkjy2021/jkkp2021/202110/t20211029_251937.html

[48] Kim JJ, et al. Suicide risk among perinatal women who report thoughts of self-harm on depression screens. Obstet Gynecol. 2015;125(4):885–93. 10.1097/AOG.0000000000000718 25751206

[49] Pope CJ, et al. A prospective study of thoughts of self-harm and suicidal ideation during the postpartum period in women with mood disorders. Arch Womens Ment Health. 2013;16(6):483–8. 10.1007/s00737-013-0370-y 23784481

[50] Chen C, et al. The diagnostic accuracy of the Edinburgh Postnatal Depression Scale without the self-harm item. J Psychiatr Res. 2023;165:70–6. 10.1016/j.jpsychires.2023.07.015 37478685

[51] Postpartum Depression: Action Towards Causes and Treatment (PACT) Consortium. Heterogeneity of postpartum depression: a latent class analysis. Lancet Psychiatry. 2015;2(1):59–67. 10.1016/S2215-0366(14)00055-8 PMC4800818 26359613

[52] Santos HJ, Tan, Salomon R. Heterogeneity in perinatal depression: how far have we come? A systematic review. (1435 – 1102 (Electronic)). 10.1007/s00737-016-0691-8 PMC5507213 27796597

[53] Waqas A, Nadeem M, Rahman A. Exploring Heterogeneity in perinatal depression: a comprehensive review. (1471-244X (Electronic)). 10.1186/s12888-023-05121-z PMC10478465 37667216

[54] Carona C et al. Mental health profiles of women at high-risk for postpartum depression: a latent profile analysis. Int J Mental Health, 2023. 52.

[55] Sharp M, et al. Internalized Weight Bias, Weight-Related Experiences, And Peripartum Weight. J Midwifery Womens Health. 2023;68(4):490–8. 10.1111/jmwh.13480 PMC10497705 36912269

[56] Deol S et al. Intersecting Expectations when Expecting: Pregnancy-Related Weight Stigma in Women of Colour. J Immigr Minor Health, 2024. 10.1007/s10903-024-01619-0 39017811

[57] Alimoradi Z, et al. Weight-related stigma and psychological distress: A systematic review and meta-analysis. Clin Nutr. 2020;39(7):2001–13. 10.1016/j.clnu.2019.10.016 31732288

[58] Hayward LE, Vartanian LR, Pinkus RT. Weight Stigma Predicts Poorer Psychological Well-Being Through Internalized Weight Bias and Maladaptive Coping Responses. Obes (Silver Spring). 2018;26(4):755–61. 10.1002/oby.22126 29427370

[59] Easter A, et al. Antenatal and postnatal psychopathology among women with current and past eating disorders: longitudinal patterns. Eur Eat Disord Rev. 2015;23(1):19–27. 10.1002/erv.2328 PMC4309475 25345371

[60] Paans NPG, et al. Eating styles in major depressive disorder: Results from a large-scale study. J Psychiatr Res. 2018;97:38–46. 10.1016/j.jpsychires.2017.11.003 29175296

[61] Hirst KP, Moutier CY. Postpartum major depression. Am Fam Physician. 2010;82(8):926–33. 20949886

[62] Gibson EL. Emotional influences on food choice: sensory, physiological and psychological pathways. Physiol Behav. 2006;89(1):53–61. 10.1016/j.physbeh.2006.01.024 16545403

[63] Poreddi V, et al. Postpartum depression: Mental health literacy of Indian mothers. Arch Psychiatr Nurs. 2021;35(6):631–7. 10.1016/j.apnu.2021.09.003 34861956

[64] Corrêa H, et al. Postpartum depression symptoms among Amazonian and Northeast Brazilian women. J Affect Disord. 2016;204:214–8. 10.1016/j.jad.2016.06.026 27372408

[65] Matsumura K, et al. Education level and risk of postpartum depression: results from the Japan Environment and Children’s Study (JECS). BMC Psychiatry. 2019;19(1):419. 10.1186/s12888-019-2401-3 PMC6935197 31882000

[66] Yang X, et al. Maternal postnatal confinement practices and postpartum depression in Chinese populations: A systematic review. PLoS ONE. 2023;18(10):e0293667. 10.1371/journal.pone.0293667 PMC10615300 37903136

[67] Fan R. Which care? Whose responsibility? And why family? A Confucian account of long-term care for the elderly. J Med Philos. 2007;32(5):495–517. 10.1080/03605310701626331 17924274

[68] Bedford O, Hwang Kk. Guilt and Shame in Chinese Culture: A Cross-cultural Framework from the Perspective of Morality and Identity. J Theory Social Behav. 2003;33:127–44.

